# Quantitative Methodologies to Dissect Immune Cell Mechanobiology

**DOI:** 10.3390/cells10040851

**Published:** 2021-04-09

**Authors:** Veronika Pfannenstill, Aurélien Barbotin, Huw Colin-York, Marco Fritzsche

**Affiliations:** 1 Kennedy Institute for Rheumatology, University of Oxford, Roosevelt Drive, Oxford OX3 7LF, UK; veronika.pfannenstill@kennedy.ox.ac.uk (V.P.); aurelien.barbotin@dtc.ox.ac.uk (A.B.); 2 Rosalind Franklin Institute, Harwell Campus, Didcot OX11 0FA, UK

**Keywords:** mechanobiology, biomechanics, force, immune response, quantitative technology

## Abstract

Mechanobiology seeks to understand how cells integrate their biomechanics into their function and behavior. Unravelling the mechanisms underlying these mechanobiological processes is particularly important for immune cells in the context of the dynamic and complex tissue microenvironment. However, it remains largely unknown how cellular mechanical force generation and mechanical properties are regulated and integrated by immune cells, primarily due to a profound lack of technologies with sufficient sensitivity to quantify immune cell mechanics. In this review, we discuss the biological significance of mechanics for immune cells across length and time scales, and highlight several experimental methodologies for quantifying the mechanics of immune cells. Finally, we discuss the importance of quantifying the appropriate mechanical readout to accelerate insights into the mechanobiology of the immune response.

## 1. Introduction

The development of novel quantitative technologies and their application to outstanding scientific problems has often paved the way towards ground-breaking biological findings. This can be exemplified by both DNA-sequencing techniques and mass spectrometry that have in their own right transformed biological research and led to important insights into biochemical signalling pathways, cellular organization and the tissue microenvironment [1,2]. In recent years, biophysical parameters, such as molecular diffusion rates, cell material properties and mechanical force generation, have become increasingly recognized as functionally relevant for biology across various length and time scales [3,4]. Although recent advances in spectroscopy and microscopy have enabled a whole set of new biophysical studies, there remains a lack of quantitative methodologies with sufficient sensitivity to determine the wide range of biomechanical parameters relevant to the function of the human immune response at all scales, from single molecules, to cells and tissues [5,6,7].

Biomechanics focuses on the application of mechanical concepts to biological systems [8]. This includes the study of the mechanical properties of biological systems, the biological mechanisms by which these properties are regulated, as well as the understanding of how biological systems generate and respond to mechanical force [9]. Characterizing the mechanical behaviour of living cells is particularly demanding due to their heterogeneous and complex architecture, as well as their dynamic nature, which can be exemplified by the constant rearrangement of their internal components and of their surroundings [10,11,12]. Moreover, these processes involve highly interconnected signalling mechanisms, making cellular mechanical properties and force generation not only length-scale but also time-scale dependent. For example, experiments on immune cells have shown that mechanical force can alter the binding rates of individual biomolecules, such as the T-cell receptor (TCR), highlighting the importance of mechanics down to the nanoscale [13,14,15,16,17]. In this context, quantifying how immune cells continuously adjust their biomechanics to respond to a diverse mechanical tissue microenvironment represents a formidable challenge.

Building on the foundations of biomechanics, the emerging field of mechanobiology aims to uncover how intra- and extracellular mechanics are integrated into cell and tissue function and behaviour, elucidating the relationship between biomechanics and physiology [2,18]. The field represents an integral part of ongoing work that seeks to understand how cells integrate and exploit biophysical mechanisms in concert with genetics and biochemical signalling information across scales [5,19,20]. T cells, key mediators of the adaptive immune response, carry out their function in mechanically diverse and dynamic tissue microenvironments, being continuously subjected to mechanical forces generated within their surroundings [3,7]. In this regard, it is thus remarkable how T cells maintain exquisite antigen sensitivity [21,22]. Crucially, the function of immune cells has been shown to depend both on the stiffness of their environment and mechanical forces applied to immune cell receptors [23,24,25,26,27,28]. Owing to this, mechanobiology is gaining increasing interest within the context of the human immune response, but fully elucidating the likely complex role of mechanics in immune cell function relies on the robust quantification of the relevant mechanical parameters with sufficient sensitivity [29]. This represents a significant challenge, given the multi-scale nature of immune cell biomechanics and the tissue microenvironment [3].

In this review, we discuss the biological significance of mechanics for immune cells across the length and time scales that define their function, from single receptor–ligand interactions on the nanometer–sub-second scale to cell morphological changes driven, for example, by the actin cytoskeleton on the micrometer–minute scale, to the overall mechanical properties of the tissue environment. We highlight several mechanical parameters and experimental methodologies for quantifying the mechanics of living immune cells. Finally, we discuss the importance of quantifying the appropriate mechanical readout(s) to accelerate insights into the mechanobiology of the immune response.

## 2. Mechanics of Immune Cells

**Figure 1:**
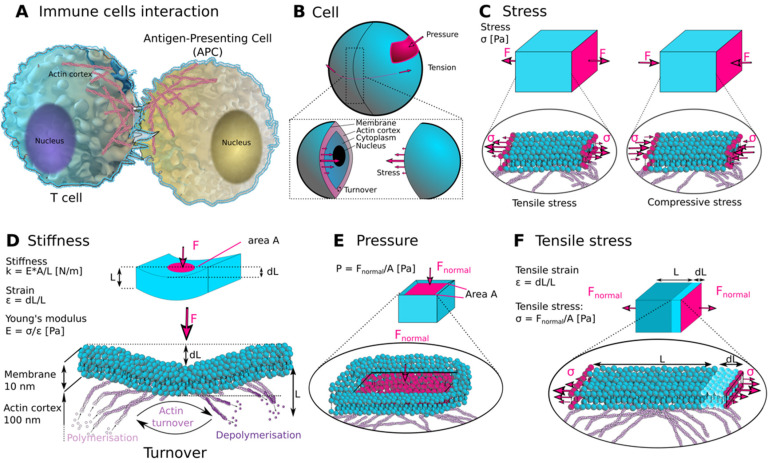
(**A**) Schematic of the interaction between a T cell and an antigen-presenting cell (APC). (**B**) Conceptual visualization of the main mechanical forces and parameters on a simplified spherical model of a cell. (**C**–**F**) Illustrations of the mechanical metrics of stress, stiffness, pressure and tensile stress on an example of a cuboidal homogenous body (upper panels) and placed into cellular context by cartoons of the membrane-cortex in the lower panels. In (**D**,**F**), the mathematical definitions for stiffness, strain, Young’s modulus, tensile strain and tensile stress are given.

Living systems, from single cells to multi-cellular organisms, can be considered to be active materials, consuming energy and maintaining a state far from thermodynamic equilibrium [30,31,32,33]. In contrast to nonbiological materials, living cells are inherently dynamic systems with the ability to generate mechanical forces and adapt their mechanical properties. For example, by dynamically regulating the organization of the cytoskeleton, cells are able to rearrange their intracellular components, such as relocating their nucleus, and generating specific architectures and protrusions, such as the lamellipodium, stress-fibres, podosomes and microvilli [33,34]. These dynamic rearrangements contribute to defining the mechanical properties of cells and their ability to generate mechanical force [33,35]. From the viewpoint of physics, mechanical force is always present when living cells undergo any interaction that changes the motion of their body or of their inner workings (Figure 1). The dynamics and interactions of cellular components are profoundly interwoven and thought to be self-reinforcing, making a theoretical mechanical description of cells and their physical microenvironment challenging. Nevertheless, a full parametrization of cellular mechanics and their surroundings is required to further our understanding of mechanobiology. A variety of different mechanical parameters has been identified that could be used to characterize the mechanical behaviour of cells (Figure 1B) [29]. The great challenge is not only to identify the control parameter(s) to describe cell mechanics but also to determine which of the parameters cells themselves depend on to regulate and adapt their functions.

The measurements of mechanical force generation and the mechanical properties of biological materials are length- and time-scale dependent. This is due to the dynamic nature of cells and their structural heterogeneity where different constituents of the cell, e.g., nucleus, cytoplasm, cytoskeleton or plasma membrane, contribute at different scales to their mechanical behaviour. Thus, it is essential to differentiate between the macroscopic, or “bulk”, mechanical properties of cells and their microscopic subcellular mechanical properties. Moreover, both the macroscopic and microscopic mechanical properties of cells as well as the forces they generate are time dependent, because their dynamics rely on a constant re-arrangement and molecular turnover [10,36,37]. Some processes, for example, actin turnover, occur at the time scale of seconds [10], while others, such as the re-location of the nucleus of activating T cells, may take multiple hours [38,39,40]. Consequently, it is essential to consider the diversity of time scales that define the mechanical behaviour of immune cells.

Typically, materials can be characterized by a series of different mechanical metrics, such as stiffness, tension, pressure and stress (Figure 1B–F). Conventionally, material properties can be described by a set of elastic and dynamic moduli, which describe the material response to external mechanical force stimuli [41]. The elasticity of a material is quantified by three main elastic moduli: Young’s modulus, shear modulus and bulk modulus. Their definitions are similar and differ primarily only by how the force is applied to the object. Among the most commonly discussed parameters in the context of cells is the Young’s modulus. The Young’s modulus E (nits: N/m^2^ or Pa), also often simply called elasticity, is an intrinsic property of the material. It is defined as tensile stress σ over tensile strain ε, where stress corresponds to the force normally applied to the cross-sectional area (given in N/m^2^ or Pa), while strain is a unitless and dimensionless quantity that describes the ratio of change in deformation to its original shape (ε=ΔL/L) (Figure 1B,C,F). The shear modulus G, also sometimes referred to as modulus of rigidity, is defined as shear stress over shear strain, and describes the tendency of a material to deform when a force is applied parallel to one of its surfaces, while the opposite surface stays at rest or experiences a counter force, such as friction. The bulk modulus K is a measure of the material’s volumetric elasticity, meaning its resistance to compression when it is uniformly loaded with a force in all directions. It is defined as volumetric stress over volumetric strain. The bulk modulus is seldomly discussed in the context of biological materials, due to their large amount of water which is incompressible. These moduli are dependent on one another, and in simple cases, they can be related via the Poisson’s ratio. For example, an ideal isotropic linear elastic material can be fully described by the Young’s modulus and the Poisson’s ratio. The Poisson’s ratio ν is a measure of the deformation of an object perpendicular to the applied load and is defined as the negative ratio of transverse strain to axial strain ($\nu =-d\varepsilon_{trans}/d\varepsilon_{axial}$). This ratio is essential in order to correctly calculate how stresses propagate through a material and is particularly important to consider when studying systems where the volume of the sample is not conserved under an applied load [42]. For example, it corresponds to a value of 0.5 for materials where the volume is conserved, while it is less than 0.5 for materials that are compressible. Most cell mechanics measurements assume that the Poisson ratio is around 0.5, but the incompressible nature of cells becomes only visible at high mechanical frequencies, as recent work has demonstrated that the cytoplasm of living cells can behave as a poroelastic material [32]. Further, the Young’s modulus, an intrinsic property of a material, should not be confused with stiffness (Figure 1D). Stiffness expresses the resistance of an object to an applied force and is measured in N/m. Although it is generally true to assume that the higher the Young’s modulus, the stiffer the material, stiffness also takes the object’s geometry into account. Stiffness is usually characterized by the object’s spring constant k, which is proportional to E. This difference is, for example, regularly applied in micropillar arrays where the stiffness of the pillar can be altered simply by changing the height or the diameter of the pillar while still using the same material with the same Young’s modulus [43]. It is also important to note the distinction between stress and pressure, as they have the same units ((N/m^2^ or Pa); Figure 1E,F). Pressure is the magnitude of the normal component of a force (external or internal) per surface of an object over which the force is applied, while stress develops inside the material and can consist of perpendicular and tangential components, and may be tensile, compressive or shear, depending on the direction of the applied load. Stress can be different at any point inside the material and is described by a tensor. As the cell cytoplasm can be considered to be a fluid, osmotic pressures associated with liquids acting on the inner cell surface need to be taken into account [44].

The Young’s modulus, the Poisson’s ratio and the mechanical stiffness of immune cells and their environment are, therefore, of great biological significance, defining how immune cells interact with their environment. It has, for example, been shown that the stiffness of the substrate can influence the efficiency of T-cell activation [23,24]. More recently, it has been shown that the stiffness of the microenvironment can regulate the activity of T cells [27]. In addition, because the mechanical properties of the cell can affect how mechanical forces are transmitted, processes such as TCR-peptide-major histocompatibility complex (pMHC) binding, which have been shown to be force sensitive, may show distinct behaviour within mechanically diverse environments [28].

The above discussion is valid for solids, yet, most biological systems, including cells, are so-called viscoelastic materials, because they exhibit time-dependent mechanical properties and thus have both elastic and viscous characteristics. Such materials can be theoretically described as a combination of an elastic solid and a viscous liquid. The viscosity of a fluid is the resistance of a liquid to deformation under a given load rate and is measured in Pascal * seconds [41,42]. The time-dependent viscoelasticity of a material is given by the storage modulus and the loss modulus [41,42]. Viscoelastic behaviour is usually measured by following the time evolution of the induced stress or strain under a constant force or by applying an oscillating force at various frequencies [41].

The viscoelastic material properties of immune cells and of the tissue microenvironment are biologically crucial, primarily owing to the time varying nature of the molecular processes that define their mechanics. The ability of immune cells to sense or adapt to their mechanical surroundings arises from the time scales at which mechanical force exerted on or by immune cells dissipates through their cellular components, for example, the plasma membrane and the actin cytoskeleton. This is, for example, important during migration where T cells change their migration modality from rolling, to integrin-mediated migration, and to squeezing, depending on the movement across the vascular wall, the lymph node, the spleen or injured tissue [45]. Thus, the specific time scales associated with particular migration modalities will influence their response to mechanical stimuli.

Specific components of the cell cytoskeleton, for example, the actin cytoskeleton, can be considered active materials [33]. The molecular constituents of the actin cytoskeleton are in constant flux resulting from their binding and unbinding. The actin turnover rate of the specific components, for example, monomeric actin, determines the structure and hence the mechanical properties of the system [9]. Using methods such as FRAP has revealed how the actin filament length-distribution influences the mechanics of the actin cortex [9]. Thus, turnover is also considered as an essential parameter in the context of the full mechanical description of the cell. An important consequence of the active nature of the actin cytoskeleton is the generation of cortical tension, a key property in regulating cell shape. Consequently, cortex tension is intimately linked to the tension within the plasma membrane. Both cortex and plasma membrane tension can be understood as the force required to deform the membrane or the cortex and have units of N/m (Figure 1B) [46]. Tension in the cortex is a direct consequence of the activity of myosin motors, with increased motor activity leading to an increase in contractility. In the plasma membrane, tension can be influenced by osmotic pressure, or the abundance of lipids or locally by the action of the actin cytoskeleton. The interconnections between the membrane and cortex lead to a high degree of coupling within their respective tensions. Interestingly, because tension can be both a local and global property, it has been hypothesized to act as a rapid mode of cellular signalling in response to external signalling; for example, in neutrophils, membrane tension has been shown to regulate cell polarity and migration [47,48].

A variety of technologies have been developed to investigate the mechanical properties of biological systems at different scales (Table 1 and Table 2) [49]. At the cellular and subcellular level, the available methodologies can be roughly divided into techniques that directly probe cell mechanical responses by indenting or deforming the cell surface or the whole cell (Table 1) and techniques that investigate subcellular properties inside cells by studying the dynamics of molecules, injected particles or beads as well as using molecular probes (Table 2). Some of the most prominent examples of direct measurement techniques are Atomic force microscopy (AFM), optical and magnetic tweezers (partial and whole cell deformations) and magnetic twisting cytometry [42,50,51,52]. For whole cell deformations, parallel-plate rheology, micropipette aspiration, shear flow methods and various cell stretching devices have been employed [42,50,51,52,53]. With some of these techniques, for example, AFM or optical tweezers, it is possible to perform static as well as dynamic measurements [32,41,42,54]. In static measurements, a constant force is applied to the sample, while in dynamic measurements, the cell is probed with oscillating forces at various frequencies. These dynamic measurements allow the quantification of time-dependent properties, such as the dynamic modulus, which is used to characterize viscoelastic behaviour. Nevertheless, possible mechanical adaptation by cells, such as stiffening or softening in response to an applied force, should be considered. For example, a study revealed changes in the elastic modulus by almost an order of magnitude upon increasing the tension on the cell [55,56].

To quantify subcellular mechanics, passive and active particle-tracking microrheology (PTM) is commonly used, where the so-called tracer particles, which can vary in size, are injected into the cell, and their motion is then followed by an optical microscope. In passive PTM, the particles’ dynamics are exclusively driven by thermal fluctuations, while in active PTM, an additional external (oscillating) magnetic force is applied to drive the particles’ movement [57,58]. Active PTM is well suited for rigid materials, where the purely thermal motion of the particles would be too small to detect [57]. What makes PTM a particularly attractive method is that it is the only well-established technique that allows the quantification of mechanical force within living cells.

Recent work by Kwapiszewska et al. used PTM in combination with two different fluorescence correlation spectroscopy (FCS) modes to quantify the mechanical properties of cells, such as the nanoscale viscosity of the cytoplasm [59,60]. Interestingly, the study revealed length-scale-dependent viscosity profiles, which were mostly independent of the cell type, as well as the origin of the cells, such as age, gender, disease or tissue. For short-length scales below 1 nm, the authors report a viscosity of about two times the viscosity of water, while when probing larger radii (e.g., >20 nm), the viscosity is about 10 × that of water. This leads to the interpretation that the cytoplasm behaves as a liquid on short-length scales (below 100 nm) and more as a gel-like structure on longer length scales (above 100 nm), which is also in agreement with results obtained previously by AFM [32]. Nevertheless, the injection of exogenous particles may alter the physical properties of the cell material under investigation. In this sense, Caragine et al. presented an interesting alternative non-invasive approach to investigate the material properties of the nucleoplasm by studying the surface dynamics and fusion kinetics of naturally occurring nucleoli in live human cells [61]. The authors reported an average value for the nucleoplasm’s viscosity of 3000 Pa.s., which corresponds well to that established previously by microrheology studies (25–1000 Pa.s). Their analysis suggests that these fusion events, although embedded in an active material, can be described as passive liquid droplets with a low surface tension fusing in a highly viscous passive liquid.

A newly emerging non-invasive technique is Brillouin microscopy that makes use of the interaction of light with traveling density fluctuations (Table 2). In solid state physics, these fluctuations are associated with acoustic waves [62]. By measuring the frequency shift of the inelastically scattered light, the material’s mechanical properties are extracted. It is important to note that Brillouin microscopy probes the material in the GHz frequency range, which is in contrast to conventional methods, such as AFM, that usually work in the MHz range, making it challenging in general to compare the measured values obtained by Brillouin microscopy to those obtained with more established methods [62]. An important advantage of Brillouin microscopy is that it is not only non-invasive and contact-free but also enables the mapping of mechanical properties in living specimens in three dimensions with a diffraction-limited spatial resolution [63]. Although some technical challenges remain, Brillouin microscopy has shown itself as a promising novel tool for quantitative biomechanical measurements [64,65]. If successful, it could become a non-invasive methodology to quantify mechanical properties intravitally on multiple-length scales and time scales, from subcellular organelles to single cells over tissue to entire organisms.

Finally, it should be noted that there is remarkable development in novel environment-sensitive fluorescent dyes, known also as functional probes (Table 2) [6]. Functional probes report on changes in the physical properties in their local microenvironment, such as changes in membrane tension, curvature or viscosity, either by changing their fluorescent lifetime or by changing their emitted fluorescent spectrum. For example, such probes have been successfully used to monitor the viscosity of mitochondria in living cells [66,67,68].

In addition to probing the mechanical properties of immune cells, understanding the precise mechanisms by which mechanical force is generated, as well as uncovering their functional significance for the immune response is of critical importance and relies on technologies that are able to quantify the resulting stresses at sufficient sensitivity [69]. Traction force microscopy (TFM) has been widely applied to measure the stresses generated during cell–substrate interactions [25,26,70,71,72]. Using an elastic substrate loaded with fluorescent beads that serve as fiducial markers, imaging the displacement of the substrate under the applied load reports on the cell generated forces. TFM has been applied in 2D, where cells adhere to a substrate, and in 3D, where cells are embedded within an elastic matrix [25,26,73]. Recent efforts also used functionalized hydrogel beads to quantify T-cell-generated forces in 3D [74]. A related approach makes use of elastic micropillars, in which elastic deformation under the influence of cellular forces can be imaged [43]. These methods provide an efficient means of quantifying the forces that cells exert on their local environment, and have provided key insights into many cellular processes, including immune cell activation [21]. In addition to TFM, in recent years, great strides have been taken to quantify force generation at the single molecule level in immune cells. There now exist several methods that allow the measurement of forces experienced by single ligand–receptor interactions or allow the application of mechanical force at the single molecule level. Molecular tension sensors consisting of engineered molecules that can undergo a force-induced confirmational change, such as a DNA hairpins, and a dye–quencher pair, show force-induced fluorescence, providing an elegant means to localize forces to individual molecules [75,76,77,78,79,80]. Biomembrane force probes and AFM have been applied to study the influence of applying a well-defined mechanical force to immune cell receptors. Such experiments have indicated that the TCR–pMHC interaction can behave as a catch bond, whereby increased the mechanical load on the receptor leads to an increase in bond lifetime. This has been suggested as a means of increasing the dynamic range of antigen discrimination. Furthermore, AFM experiments exposing single TCRs to mechanical load have induced a signalling response via calcium release, demonstrating the potential for forces alone to induce immune signalling [13,15,16,17,28,81,82,83].

**Table 2:** Some of the most common methods to measure mechanical properties in biomechanics and mechanobiology that do not rely on a direct physical contact with cells linked to the measured mechanical readout.

Technique	Measured Mechanical Parameter	Advantages	Disadvantages	Refs
Particle-tracking microrheology (PTM)	Viscoelastic material properties Force generation	Subcellular mechanical properties and force only Passive and active PTM	Invasive through injected particles	[34,36,41,57,58,59]
Fluorescence correlation spectroscopy (FCS)	Viscoelastic material properties	Very local measurement of intracellular molecules (nm) No addition of invasive particles required High time resolution (µs)	Only local information	[59,60]
Brillouin microscopy	Young’s modulus Stiffness Viscoelastic material properties	Only non-invasive contact-free method Very broad range of length and time scales (ns), from subcellular properties to entire organisms 3D method Label-free	Hard to compare to currently well-established techniques Complex data analysis	[62,63,64,65]
Environment-sensitive fluorescent dyes–functional probes	Membrane tension Viscosity	Subcellular properties Change fluorescent emission spectrum or fluorescent lifetime depending on their environment Can be easily combined with commercially available microscopes	Limited functional probes available for only particular mechanical parameters Some dyes have a very broad emission spectrum that makes it then hard to combine with the simultaneous quantification of labelled cellular structures	[6,66,67,68,75,76,77,79,80,83]

The above discussion illustrates the great diversity of methodologies that have been used to study cell material properties and cellular mechanical force generation. Although many of these methods intend to probe the same mechanical parameter(s), it is important to keep in mind that they often yield quantitatively different experimental values. For example, the Young’s modulus of cells can vary from around 100 up to 10,000 Pa [41,55], while viscosity varies by about 100-fold between measurements [42], depending on the applied technique and its sensitivity. Sometimes, these variations have been thought to be attributed to biological diversity, such as cell type or cell culture conditions, for example, cell passage number, variations in temperature or differences in pH, but not always. Comparing experimental values obtained by different techniques is not trivial, primarily because the mechanical response of cells strongly depends on the applied force profile, which can vary substantially between the techniques [42]. For example, probing a cell at a given force with a micron-scale spherical AFM cantilever will produce a very different mechanical response to the same force applied using a sharp tip that can probe details of the sub-network structure. This issue was recently addressed by a collaborative effort of several laboratories who performed mechanical measurements using the most widely applied techniques on the very same cell line, minimizing biological variations [42]. Their analysis showed that, although biological variations were minimized, the average values for the measured elastic and dynamic moduli still vary by at least two orders of magnitude. Their work highlights that certain cell mechanical techniques should be applied rather complementarily, such as AFM and PTM, since they are probing mechanical properties with fundamentally different force profiles as well as at different length and time scales.

## 3. Discussion and Conclusions

In this review, we highlighted how recent advances in the development and application of quantitative methodologies will transform our understanding of immune cell mechanobiology in the years to come. Quantifying both mechanical force production and mechanical properties is of critical importance for the understanding of active materials. Any material can be described by a full parametrization of its mechanical properties, but the identification of the right mechanical metric justifying its responses to interactions with the external environment is challenging. This is of critical relevance for cells of the immune system, for example, T cells, which are highly dynamic and employ active force generation for antigen recognition and for cytotoxicity in mechanically diverse environments without losing their function [21,22]. Thus, to better understand the immune response, a mechanical description of cells and a proper quantification of their mechanical parameters are required [29]. Aiding in the understanding of different mechanical metrics, we highlighted the most common readouts, such as mechanical force, stiffness, stress, strain, pressure, tension, Young’s modulus, viscosity and Poisson’s ratio, in a cellular context. Moreover, we discussed these parameters in the framework of the active nature of immune cells, including the cytoskeletal actin turnover, actin cortex and membrane tension. In light of the discussion, it is also important to note that these parameters derived from classical mechanics are conceptually compatible with those at cellular length scales and time scales. As cells are mechanically heterogeneous, the propagation of stress and strain throughout cells depends to a large extent on the molecular connections, for example, between the plasma membrane and the cytoskeleton, or other organelles, such as the nucleus [84,85]. Thus, one must carefully consider whether the described mechanical metrics hold when linking them to their molecular origin and dynamics; for example, careful consideration of the dynamic nature of the actin cytoskeleton led to the theoretical model of active gel theory [86].

Although we did not cover the entire spectrum of possible mechanical readouts, parameters and cell mechanics techniques, we provided the reader with an overview of the most commonly measured mechanical parameters in living cells, linking each of them to possible measuring techniques that enable a robust quantification. We also highlighted the current challenges when comparing experimental values obtained with different methodologies in terms of biological significance, data analysis and theoretical assumptions. Common to the quantification of both cellular mechanical properties and force generation is the need to experimentally directly interact with the cell. With the exception of Brillouin microscopy [63], the majority of methods expose the living system to some mechanical engagement, from AFM indentation to the stiffness of the substrate applied using TFM. Given this requirement, consideration should be given to the active nature of the biological system, for example, during AFM indentation, the cell may actively adapt its biomechanics in response to the initial indentation, inducing a softening or stiffening compared to the unperturbed stiffness. Similarly, in TFM, the mechanical feedback resulting from the substrate stiffness may result in an increase or decrease in measured stress [87]. Another important issue in this context is throughput. It is well known that cell signalling and cell responses can be very heterogenous under the same experimental conditions. Thus, ensuring a statistical robustness in the results is crucial. This is the advantage of Brillouin microscopy as well as several recently developed microfluidic-based methods, which are contact-free, non-invasive and allow high-speed measurements of 10–10,000 cells per second [88,89].

In our view, the ongoing combination of three complementary scientific approaches will further our understanding of mechanobiology: (i) the development of quantitative technologies with the right sensitivity, also enabling correlative and simultaneous mechanical measurements across scales; (ii) in vitro reconstitution as well as mechanical measurements of model systems that allows a better characterization of particular cellular components; (iii) advances in theoretical modelling addressing specifically the multi-scale problem of the mechanobiology of living cells. Despite the remarkable progress in advancing cell mechanics technology and identifying relevant mechanical parameters, solving the formidable challenge of how cells integrate mechanical cues into function remains. The key to this puzzle lies in understanding how the acquired genetic, biochemical and biophysical features feed into one another. One promising experimental approach is to further develop simultaneous measurement techniques. For example, quantifying both cell mechanical properties and signalling events simultaneously might yield a more complete picture of how mechanics, force generation and biochemical signalling are coupled across scales, from single receptor–ligand interactions to cell morphological changes driven by the actin cytoskeleton, to the overall mechanical properties of the tissue environment. Recent efforts of combining AFM with TFM as well as with different fluorescence imaging approaches have already proven to be promising [36,87,90,91,92,93]. Quantifying the mechanobiology of the immune response utilizing methodologies with the appropriate sensitivity may thus be the route to enhancing our understanding of the role of mechanobiology in health and disease.

## Figures and Tables

**Table 1:** Some of the most commonly used techniques to measure mechanical properties in biomechanics and mechanobiology that are based on a direct mechanical contact with the cells linked to the measured mechanical readout.

Technique	Measured Mechanical Parameter	Advantages	Disadvantages	Refs
Atomic force microscope (AFM)	Young’s modulus Stiffness Viscoelastic material properties Membrane/cortex tension Force generation	Static and dynamic measurements Broad range of length and time scales, from single-molecule interactions to whole-cell deformations measured from ms to hrs Cellular and subcellular properties Well suited for studying molecular interactions with cells Piconewton resolution	Direct mechanical interaction with cells Complex analysis since the overlay of mechanical responses from various cellular components is measured	[32,36,41,42,49,50,51,52]
Optical and magnetic tweezers (OT and MT)	Young’s modulus Stiffness Viscoelastic material properties Membrane/cortex tension Force generation	Static and dynamic measurements Molecular to cellular interactions Possible stretching and twisting Piconewton range	Sample heating (OT) Need for magnetic particles (MT)	[36,41,42,49,51,54]
Micropipette aspiration/Biomembrane force probe	Young’s modulus Stiffness Viscoelastic material properties Membrane/cortex tension Pressure Force generation	Local and global cell mechanical properties Piconewton resolution Low cost	Most set ups have a low throughput Limited spatial resolution to the micron scale Direct mechanical interaction with cells Possible cell damage	[13,36,49,51,53]

## Data Availability

Not applicable.
